# Efficacy and Safety of PrabotulinumtoxinA for the Treatment of Moderate-to-Severe Glabellar Lines: A GRADE-Assessed Systematic Review and Meta-Analysis

**DOI:** 10.1093/asjof/ojag202

**Published:** 2026-10-14

**Authors:** Zain Ul Abideen Shahid, Muhammad Hassan, Syeda Verisha Batool, Mehak Faisal, Sameer Tariq, Syeda Huda Fatima, Muhammad Hussain, Syeda Jazilah Faisal, Ghulam Taha Khan, Aleena Batool, Syed Hadi Raza Rizvi, Murtaza Aslam, Talha Aamir, Huzaifa Salman Madni, Saad Ashraf, Ibrahim Nagmeldin Hassan

## Abstract

Glabellar lines are among the earliest and most visible signs of facial aging and can convey negative emotions while diminishing quality of life. PrabotulinumtoxinA is a 900 kDa formulation of botulinum toxin type A that provides targeted neuromuscular blockade to treat dynamic wrinkles through localized muscle relaxation. Individual trials have reported encouraging results, yet pooled estimates were needed to more accurately quantify efficacy and safety. The objective of this meta-analysis was to evaluate the efficacy and safety of prabotulinumtoxinA for the treatment of glabellar lines. The authors searched PubMed, Cochrane CENTRAL, Scopus, Embase, and ClinicalTrials.gov through May 2026 and identified 3 double-blind, placebo-controlled Phase III randomized controlled trials evaluating a 20 U dose of prabotulinumtoxinA vs placebo; 3 additional studies were included for qualitative safety analysis. In pooled random-effects analyses, prabotulinumtoxinA significantly increased the likelihood of achieving none-or-mild glabellar line severity scores (GLS score 0/1) at Day 30 (risk ratio [RR] = 19.24, 95% CI, 12.08-30.64; *P* < .01), demonstrated early treatment response at Day 2 (≥1-point improvement in GLS score: RR = 3.61, 95% CI, 2.52-5.19; *P* < .01) and showed greater sustainability through Day 150 (RR = 3.47, 95% CI, 2.13-5.65; *P* < .01). Safety outcomes, including treatment-emergent adverse events and specific safety endpoints, were comparable to placebo, with no significant differences. PrabotulinumtoxinA was well-tolerated and produced substantial and sustained improvement of glabellar lines. Further large-scale, long-term trials are needed to establish its durability and long-term safety profile.

**Level of Evidence**: 2 (Therapeutic) 
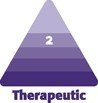

Glabellar lines, also known as glabellar rhytides, are vertical furrows that develop between the eyebrows secondary to the repetitive contraction of the corrugator supercilii, procerus, and depressor supercilii muscles. These dynamic wrinkles represent one of the earliest and most prominent manifestations of facial aging.^1^ Beyond their aesthetic significance, glabellar lines impose a substantial psychosocial burden, adversely affecting emotional communication, increasing perceived age, and diminishing self-esteem and quality of life.^2^ Studies have demonstrated that up to 85% of individuals with moderate-to-severe glabellar lines experience substantial psychological impact at baseline.^1,3^ These facial features may transmit impressions of negative emotions such as anger, anxiety, or sadness.^2^ Without intervention, these wrinkles progressively transition from dynamic to static lines, underscoring the clinical need for effective therapeutic interventions.^4^

Botulinum toxin type A (BoNT-A) has emerged as the gold standard treatment for glabellar lines and represents the most frequently performed cosmetic procedure worldwide.^3,4^ Botulinum neurotoxin type A exerts its principal local effect by binding to presynaptic cholinergic nerve terminals, undergoing internalization, and cleaving synaptosomal-associated protein 25 (SNAP-25), thereby disrupting soluable N-ethylmaleimide-sensitive factor attachment protein receptor (SNARE)-mediated synaptic vesicle fusion and blocking acetylcholine release at the neuromuscular junction. This produces temporary reduction in activity of the targeted facial muscles and consequent attenuation of dynamic glabellar lines. In addition to its neuromuscular effects, BoNT has been reported to modulate the release of sensory neuropeptides and other neurotransmitters, including glutamate, substance *P*, and calcitonin gene-related peptide, particularly from peripheral sensory nerve terminals. These additional effects may contribute to the broader biological and sensory actions of BoNT-A beyond its primary neuromuscular effects, supporting additional sensory and nonmotor mechanisms of action.^3,5,6^ Clinical effects of BoNT-A can become detectable within hours after injection, although the timing and magnitude of onset vary according to the specific formulation, injection site, dose, assessment method, and definition of clinical onset. Objective studies have demonstrated measurable effects as early as 12 hours after administration, whereas studies in glabellar lines have reported visible improvement within 24 hours and median onset generally occurring within 2 to 4 days for abobotulinumtoxinA.^3,7,8^ Multiple BoNT-A formulations have received regulatory approval for aesthetic indications, including onabotulinumtoxinA, abobotulinumtoxinA, incobotulinumtoxinA, and prabotulinumtoxinA.^4^ Meta-analytic evidence specifically evaluating a single 20 U dose of BoNT-A for glabellar lines has demonstrated high efficacy, with pooled response rates significantly superior to placebo and no significant difference in the frequency of adverse events between treatment and placebo groups.^9^

PrabotulinumtoxinA is a 900 kDa BoNT-A formulation that received US FDA approval in February 2019 for the temporary improvement of moderate-to-severe glabellar lines in adults.^10^ The development program for prabotulinumtoxinA included multiple Phase II and Phase III randomized controlled trials evaluating its efficacy, safety, duration of effect, and noninferiority compared with established BoNT-A products.^11-14^ Although individual trials have demonstrated promising results, a comprehensive systematic review of the available evidence is essential to provide clinicians with robust, pooled estimates of treatment efficacy and safety. Meta-analysis offers the statistical power to detect treatment effects with greater precision, identify rare adverse events, evaluate consistency of findings across studies, and inform evidence-based clinical decision making. Although prabotulinumtoxinA has been evaluated in several clinical studies and summarized in previous narrative and systematic reviews, a quantitative systematic review and meta-analysis focused specifically on the efficacy and safety of the FDA-approved 20 U regimen for moderate-to-severe glabellar lines remains limited.^15^ Because BoNT products are measured in product-specific biological activity units that are not interchangeable, we restricted the quantitative analysis to the approved 20 U prabotulinumtoxinA regimen rather than combining different BoNT products or nonequivalent doses. This approach was intended to provide a clinically interpretable estimate for the approved treatment regimen while minimizing heterogeneity arising from differences in product potency and dosing.

Consequently, the present systematic review and meta-analysis aims to comprehensively evaluate the efficacy and safety of prabotulinumtoxinA for the treatment of moderate-to-severe glabellar lines in adult patients. This review will provide clinicians with pooled evidence to guide treatment decisions and inform patient counseling regarding prabotulinumtoxinA therapy for facial aesthetic indications.

## METHODS

### Protocol Registration

This systematic review and meta-analysis was conducted in accordance with the Preferred Reporting Items for Systematic Reviews and Meta-Analyses (PRISMA) 2020 guidelines.^16^ The review protocol was prospectively registered with the International Prospective Register of Systematic Reviews (PROSPERO; registration number: CRD420261394860), on May 15, 2026, before formal screening of identified records.^17^

### Data Sources and Search Strategy

A comprehensive literature search was performed across PubMed/MEDLINE, Cochrane CENTRAL, Scopus, Embase, and ClinicalTrials.gov from database inception through May 31, 2026. To ensure completeness, the reference lists of all included studies and relevant review articles were manually screened for additional eligible records. No restrictions were applied regarding language or publication date.

The search strategy incorporated both Medical Subject Headings and free-text keywords relevant to the population and intervention. The core search terms included (“glabellar lines” OR “facial wrinkle” OR “glabellar wrinkle”) AND (“prabotulinumtoxinA” OR “Jeuveau” OR “botulinum toxin type A”) AND (“placebo” OR “control”). The complete database-specific search strategies are provided in Supplementary Table 1.

### Study Selection and Eligibility Criteria

All retrieved records were imported into Rayyan.AI for deduplication and screening.^18^ Two reviewers (S.T. and S.H.F.) independently screened titles and abstracts for potential eligibility, followed by full-text assessment of shortlisted studies based on predefined criteria. Discrepancies were resolved through discussion or consultation with a third reviewer (S.J.F.).

Eligibility was defined using the PICOS framework.^19^ For the primary quantitative analysis, eligible studies were randomized controlled trials enrolling adults with moderate-to-severe glabellar lines treated with prabotulinumtoxinA 20 U vs placebo.

For the qualitative safety analysis, we additionally included nonplacebo comparator trials and repeat-dose/open-label studies of prabotulinumtoxinA that reported long-term or extended safety outcomes.

### Data Extraction and Outcome Measures

Data extraction was performed independently by 2 reviewers (G.T.K. and M.F.) using a standardized, prepiloted Microsoft Excel form. Extracted variables included study characteristics (author, year, and trial name); design features, baseline participant characteristics (eg, demographics, age, and glabellar line scale glabellar line severity [GLS] at baseline), intervention and comparator details (drug, dose, and duration); and all relevant efficacy and safety outcomes. The primary efficacy outcome included achievement of none-or-mild (GLS score 0 or 1) at Day 30. Secondary efficacy outcomes comprised early and sustained GLS improvement (≥1-point reduction) at Days 2 and 150; composite ≥2-point GLS improvement at Days 30, 90, 120, and 150; Global Aesthetic Improvement Scale (GAIS) responder rates at Day 30; and Subject Satisfaction Scale (SSS) response at Day 30. Safety outcomes included any treatment-emergent adverse events (TEAEs), study drug-related adverse events, and adverse events of special interest (AESIs).

### Quality Assessment

The risk of bias of included randomized controlled trials was independently assessed by 2 reviewers (Z.U.A.S. and M.A.), using the Cochrane Risk of Bias 2 (RoB2) tool.^20^ This instrument evaluates bias across 5 domains: the randomization process, deviations from intended interventions, missing outcome data, measurement of outcomes, and selection of the reported results. Each study was judged as having low risk of bias, some concerns, or high risk of bias according to established signaling algorithms. Any disagreements were resolved through discussion or consultation with a third reviewer, and results were visually summarized using a traffic light plot.

### Statistical Analysis and Assessment of Heterogeneity

We conducted all statistical analyses using Review Manager software (RevMan, version 5.4; The Cochrane Collaboration).^21^ For dichotomous outcomes, participants were classified according to whether they experienced or achieved the prespecified event or response criterion (eg, achievement of a GLS response or occurrence of an adverse event). These outcomes were summarized as risk ratios (RRs) with corresponding 95% CIs.

To accommodate expected between-study variability arising from differences in clinical populations, dosing protocols, and trial methodologies, we employed a random-effects model (DerSimonian–Laird method) for all pooled estimates.^22^ A 2-tailed *P*-value threshold of <0.05 denoted statistical significance.

Between-study heterogeneity was evaluated using the Higgins *I*^2^ statistic, where *I*^2^ values below 25% were interpreted as low, 25%-50% as moderate, and above 50% as high heterogeneity.

### Subgroup and Sensitivity Analysis

Prespecified subgroup analyses were conducted for all primary and secondary efficacy endpoints according to assessment modality, investigator-assessed and participant-assessed (PA), to examine concordance between clinician-evaluated and patient-reported outcomes. Whenever substantial heterogeneity was present for an outcome, we planned to perform leave-one-out sensitivity analyses to examine the impact of individual studies on the overall pooled result and heterogeneity.

### Certainty of Evidence (GRADE Assessment)

We assessed the certainty of evidence for each primary outcome using the GRADE (Grading of Recommendations Assessment, Development and Evaluation) approach.^23^ This framework evaluates 5 domains: risk of bias, inconsistency, indirectness, imprecision, and publication bias. The final certainty for each outcome was rated as high, moderate, low, or very low, reflecting our confidence in the effect estimates. A detailed GRADE evidence profile with domain-specific judgments is provided in Supplementary Table 2.

## RESULTS

### Study Selection

A total of 1205 records were identified through database searching, including PubMed (*n* = 162), ClinicalTrials.gov (*n* = 136), Cochrane (*n* = 320), Scopus (*n* = 363), and Embase (*n* = 224). After removal of 645 duplicates, 560 unique records underwent title and abstract screening, of which 534 were excluded. The full texts of 26 studies were assessed for eligibility, and 20 studies were excluded for wrong study design (*n* = 12) including observational studies, reviews, post hoc analyses, case reports/series, nonrandomized studies, incorrect patient population (*n* = 5) which included studies not enrolling adults with moderate-to-severe glabellar lines, or unreported outcomes (*n* = 3) which represents studies meeting other eligibility criteria but not reporting extractable data for prespecified efficacy/safety outcomes. Ultimately, 6 studies met the inclusion criteria, of which 3 were included in the quantitative meta-analysis and 3 were included in the qualitative safety analysis only. The complete study selection process is illustrated in the PRISMA flow diagram (Figure 1).

**Figure 1.**
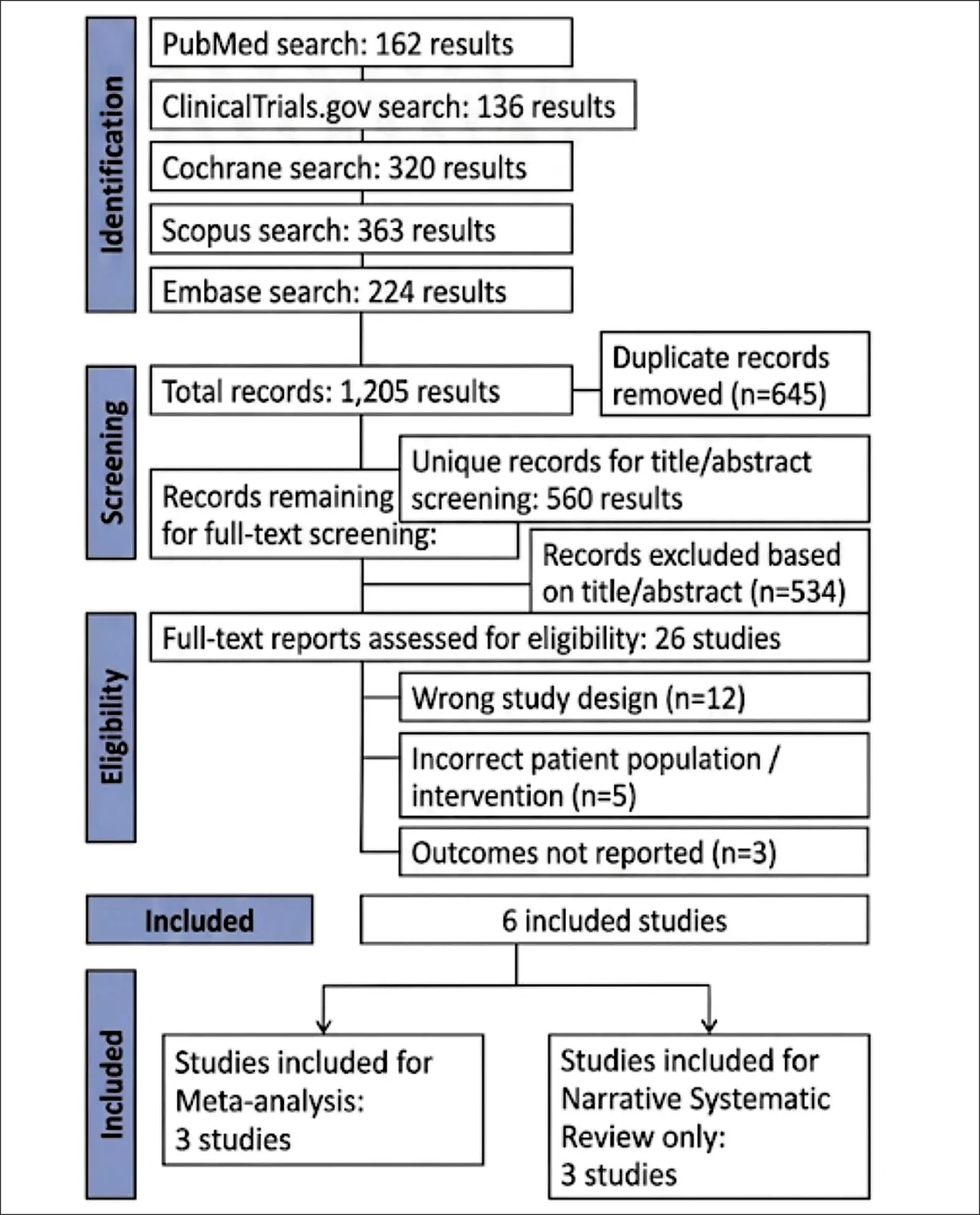
PRISMA flow diagram of study selection.

### Study and Baseline Patient Characteristics

Across 3 randomized Phase III trials (EV 001 March 2019, EV 002 March 2019, and [EVB-003]), 948 participants were included across prabotulinum (20 U) and placebo groups. Sample sizes ranged from 49 to 246 participants per study. Mean age ranged from 48.4 to 51.5 years, with female representation between 83.7% and 94.0%. The majority of participants were White, ranging from 67.3% to 88.5%, whereas Black/African American participants accounted for 1.2% to 8.3% and Asian participants for 0.8% to 4.8%. Severe glabellar line severity at maximum frown on investigator assessment ranged from 66.7% to 84.6%, whereas moderate severity ranged from 15.4% to 33.3%. Severe glabellar line severity on subject assessment ranged from 77.2% to 83.3%, with moderate severity ranging from 16.7% to 22.8%. The mean Hospital Anxiety and Depression Scale (HADS)-Anxiety scores were ∼5.2 to 5.3, and mean HADS-Depression scores ranged from 2.4 to 2.7 in the studies reporting these outcomes. Detailed study and patient baseline characteristics are provided in Table 1.

**Table 1.** Baseline Characteristics of Studies and Patients

Baseline characteristics	EV 001 March 2019		EV 002 March 2019		Rzany April 2019 (EVB-003)	
	Prabotulinum	Placebo	Prabotulinum	Placebo	Prabotulinum	Placebo
Study design	RCT Phase III		RCT Phase III		RCT Phase III	
Country	USA/10 centers		USA/10 centers		France, Germany, Sweden, UK, Canada/19 centers	
Doses of prabotulinum	20 U	Placebo	20 U	Placebo	20 U	Placebo
Follow-up duration (days)	150		150		150	
Sample size	246	84	246	78	245	49
Age (years), mean ± SD (min, max)	50.2 ± 11.76 (22, 81)	50.4 ± 11.95 (23, 74)	51.5 ± 11.54 (21, 81)	50.4 ± 10.14 (18, 71)	48.8 ± 10.73 (22, 79)	48.4 ± 10.84 (26, 71)
Age <65 years, *n* (%)	220 (89.4)	75 (89.3)	219 (89.0)	72 (92.3)	228 (93.1)	45 (91.8)
Female, *n* (%)	227 (92.3)	79 (94.0)	220 (89.4)	70 (89.7)	220 (89.8)	41 (83.7)
White race, *n* (%)	205 (83.3)	63 (75.0)	215 (87.4)	69 (88.5)	165 (67.3)	36 (73.5)
Black/African American race, *n* (%)	18 (7.3)	7 (8.3)	19 (7.7)	6 (7.7)	3 (1.2)	1 (2.0)
Asian race, *n* (%)	2 (0.8)	4 (4.8)	5 (2.0)	2 (2.6)	6 (2.4)	1 (2.0)
Other race, *n* (%)	21 (8.5)	10 (11.9)	7 (2.8)	1 (1.3)	8 (3.3)	1 (2.0)
Severe GLS at max frown IA, *n* (%)	168 (68.3)	56 (66.7)	204 (82.9)	66 (84.6)	183 (74.7)	36 (73.5)
Moderate GLS at max frown IA, *n* (%)	78 (31.7)	28 (33.3)	42 (17.1)	12 (15.4)	62 (25.3)	13 (26.5)
Severe GLS at max frown SA, *n* (%)	190 (77.2)	66 (78.6)	200 (81.3)	65 (83.3)	201 (82.0)	40 (81.6)
Moderate GLS at max frown SA, *n* (%)	56 (22.8)	18 (21.4)	46 (18.7)	13 (16.7)	44 (18.0)	9 (18.4)
Mean HADS-Anxiety Score, mean ± SD (min, max)	N/A		N/A		5.3 ± 3.20 (0, 15)	5.2 ± 2.74 (0, 10)
Mean HADS-Depression Score, mean ± SD (min, max)	N/A		N/A		2.4 ± 2.45 (0, 15)	2.7 ± 2.43 (0, 10)

GLS, glabellar line severity; IA, investigator-assessed; N/A, not applicable; SA, subject-assessed; SD, standard deviation.

### Quality Assessment of Included Studies

Two trials EV-001 and EV-002 were judged to have a low risk of bias across all domains and overall.^3^ In contrast, Rzany et al was judged to have some concerns overall, with some concerns identified in the domains of bias because of deviations from intended interventions and bias in measurement of the outcome, whereas the remaining domains were judged to have low risk of bias, as shown in Supplementary Figures 1 and 2.

### Efficacy Outcomes

#### Achievement of None-or-Mild Glabellar Line Severity (GLS Score 0/1) at Day 30

Three randomized controlled trials (RCTs) (EV-001, EV-002, and Rzany 2019) evaluated the proportion of participants achieving a GLS score of 0 or 1 at Day 30.^3,11^ Pooled analysis demonstrated a statistically significant superiority of prabotulinum over placebo (RR = 19.24, 95% CI, 12.08-30.64; *I*^2^ = 0%; *P* < .01, *I*^2^ = 0%), as shown in Figure 2.

**Figure 2.**
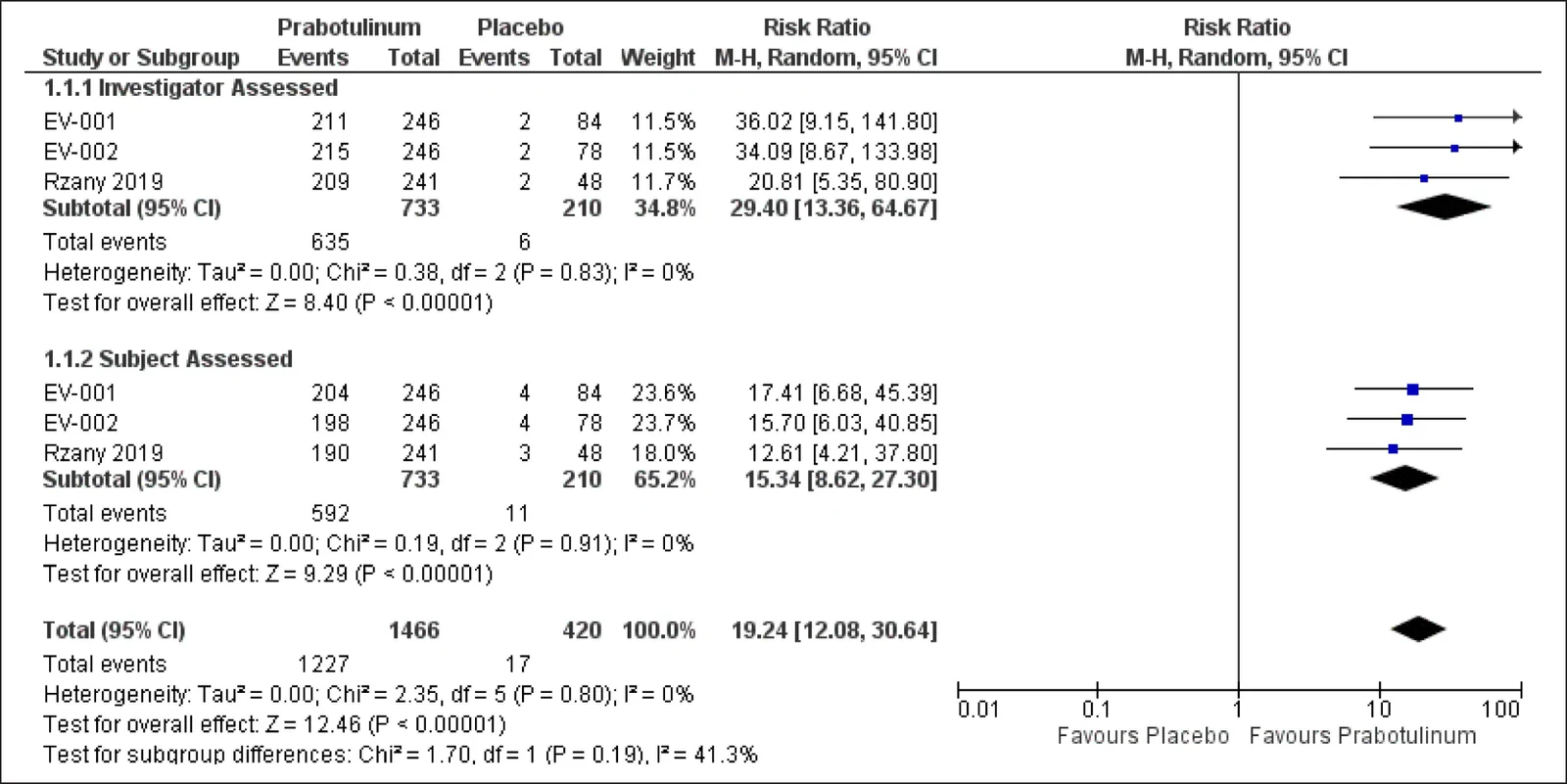
Forest plot of achievement of none-or-mild glabellar line severity (GLS score 0/1) at Day 30.

In the investigator-assessed subgroup, the treatment effect was markedly amplified (RR = 29.40, 95% CI, 13.36-64.67; *I*^2^ = 0%; *P* < .01). The SA subgroup similarly demonstrated a consistent benefit (RR = 15.34, 95% CI, 8.62-27.30; *I*^2^ = 0%; *P* < .00001). No heterogeneity was observed in either subgroup (*I*^2^ = 0%).

#### Early and Sustained GLS Improvement (≥1-Point Reduction)

Three studies (EV-001, EV-002, and Rzany 2019) assessed the proportion of participants achieving a ≥ 1-point GLS reduction at Day 2 and Day 150.^3,11^

At Day 2, pooled analysis demonstrated a significant early onset of effect (RR = 3.61, 95% CI, 2.52-5.19; *I*^2^ = 42%; *P* < .01), as shown in Figure 3. In the investigator-assessed subgroup, the effect remained significant (RR = 4.64, 95% C,L, 2.54-8.47; *I*^2^ = 52%; *P* < .01), with moderate heterogeneity (*I*^2^ = 52%) that resolved upon exclusion of EV-001 (*I*^2^ = 0%), with no attenuation of the overall effect, as shown in Supplementary Figure 3. Subject-assessed data from EV-001 and EV-002 individually yielded RRs of 2.87 (95% CI, 2.00-4.13, *P* < .01).

**Figure 3.**
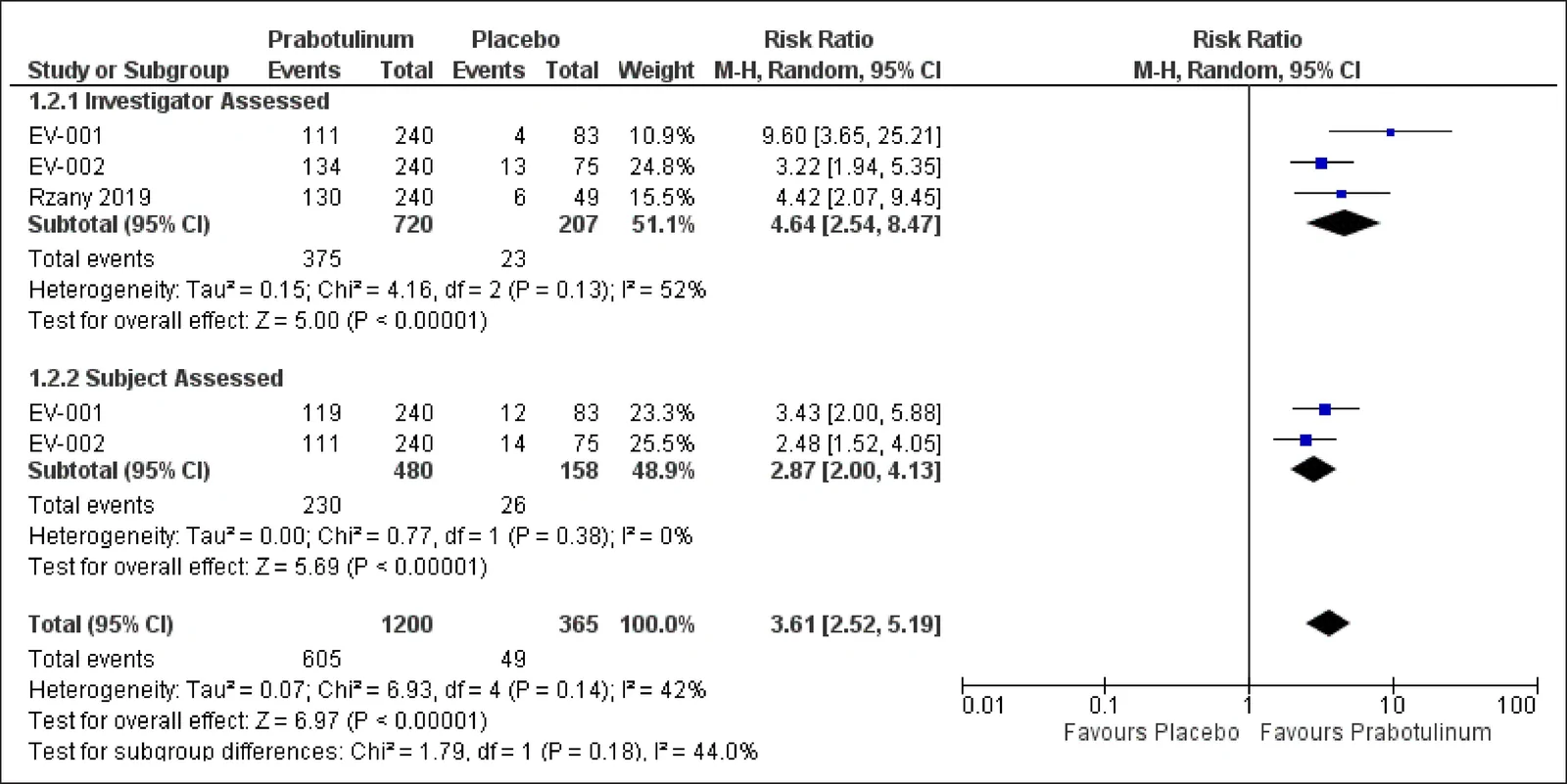
Forest plot of ≥1-point glabellar line severity improvement at Day 2.

At Day 150, prabotulinum continued to demonstrate a statistically significant advantage over placebo (RR = 3.47, 95% CI, 2.13-5.65; *I*^2^ = 60%; *P* < .00001), as shown in Figure 4. Investigator-assessed outcomes showed high response rates with no heterogeneity (RR = 5.17, 95% CI, 3.15-8.49; *I*^2^ = 0%; *P* < .00001). In the SA subgroup, significant heterogeneity was present (*I*^2^ = 54%); exclusion of EV-002 eliminated heterogeneity (*I*^2^ = 0%) while preserving the significant effect (RR = 2.33, 95% CI, 1.37-3.95; *P* < .00001), as shown in Supplementary Figure 4.

**Figure 4.**
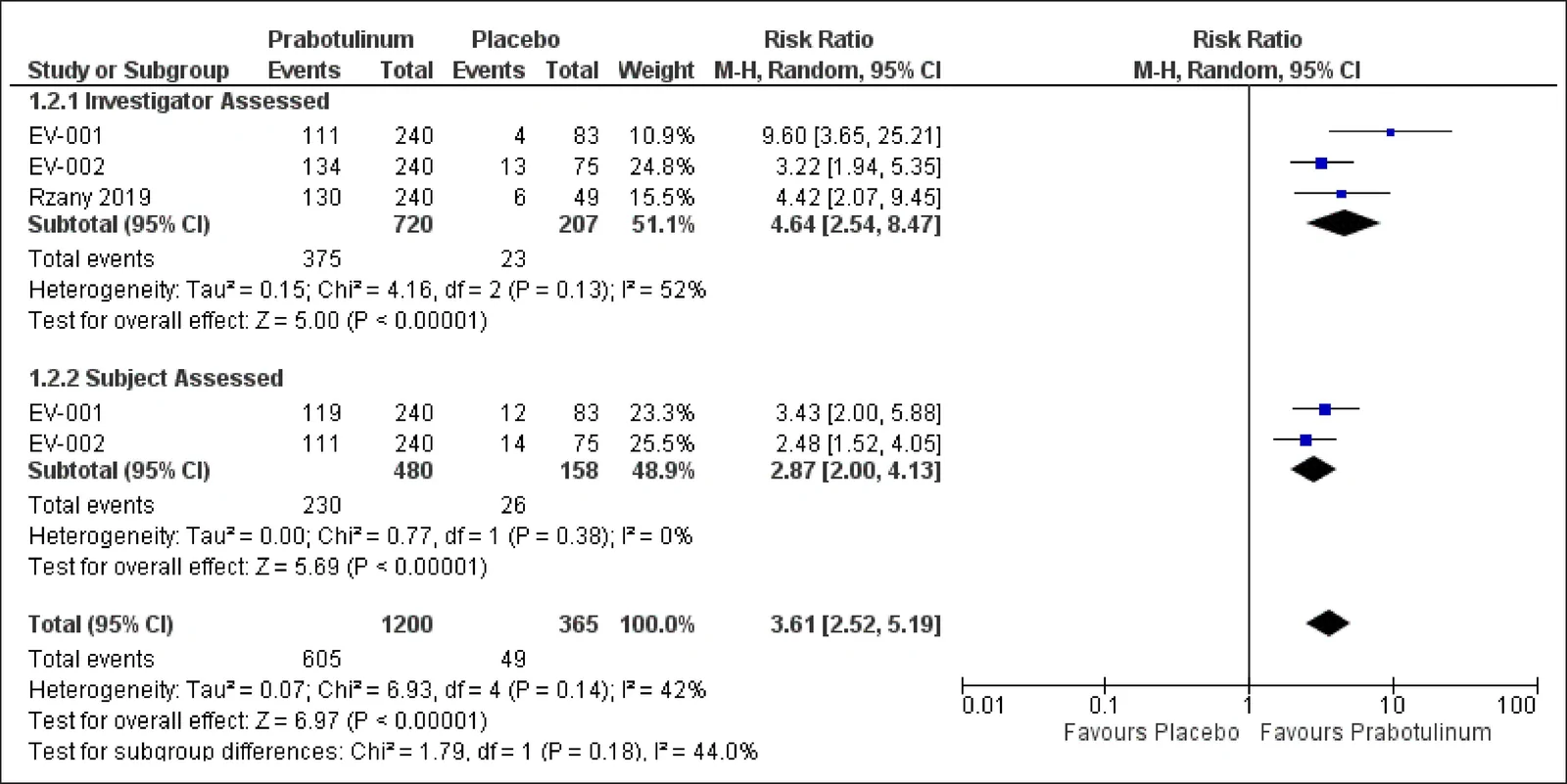
Forest plot of ≥1-point glabellar line severity improvement at Day 150.

#### Composite ≥2-Point GLS Improvement at Day 30

Two trials (EV-001 and EV-002) evaluated the proportion of participants achieving a ≥ 2-point GLS at Day 30.^3^ Prabotulinum demonstrated a markedly higher response rate than placebo, with a significant benefit of (RR = 28.83, 95% CI, 16.09-51.66; *I*^2^ = 0%; *P* < .00001), with no evidence of between-study heterogeneity. Consistent treatment effects were observed in investigator-assessed (RR = 39.40, 95% CI, 12.85-120.75), SA (RR = 20.11, 95% CI, 9.16-44.14), and composite responder analyses (RR = 54.39, 95% CI, 13.71-215), as shown in Supplementary Figure 5.

#### Composite ≥2-Point GLS Improvement at Multiple Time Points

Two trials (EV-001 and EV-002) assessed the composite endpoint requiring simultaneous IA and SA ≥2-point GLS improvement.^3^ The overall pooled estimate was (RR = 13.30, 95% CI, 4.95-35.76; *I*^2^ = 0%; *P* < .01). Time point-specific analyses consistently favored prabotulinum across all follow-up visits, with effect sizes attenuating predictably over time: Day 90 (RR = 26.15, 95% CI, 5.28-129.52), Day 120 (RR = 9.51, 95% CI,: 1.88-48.13; *P* = .006), and Day 150 (RR = 7.73, 95% CI, 1.05-56.77; *P* = .04). No between-study heterogeneity was observed at any time point (*I*^2^ = 0%), as demonstrated in Supplementary Figure 6.

#### GAIS Responder Rate at Day 30

Three trials (EV-001, EV-002, and Rzany 2019) assessed GAIS responder rates at Day 30.^3,11^ Pooled analysis demonstrated a significantly higher proportion of responders with prabotulinum (RR = 12.05, 95% CI, 8.55-16.99; *I*^2^ = 0%; *P* < .00001). In the investigator-assessed subgroup, the effect was substantially larger (RR = 13.66, 95% CI, 7.02-26.59; *I*^2^ = 35%; *P* < .01), with low and nonsignificant heterogeneity. Subject-assessed responses were consistent and homogeneous (RR = 11.67, 95% CI, 7.29-18.67; *I*^2^ = 0%; *P* < .01), as shown in Figure 5.

**Figure 5.**
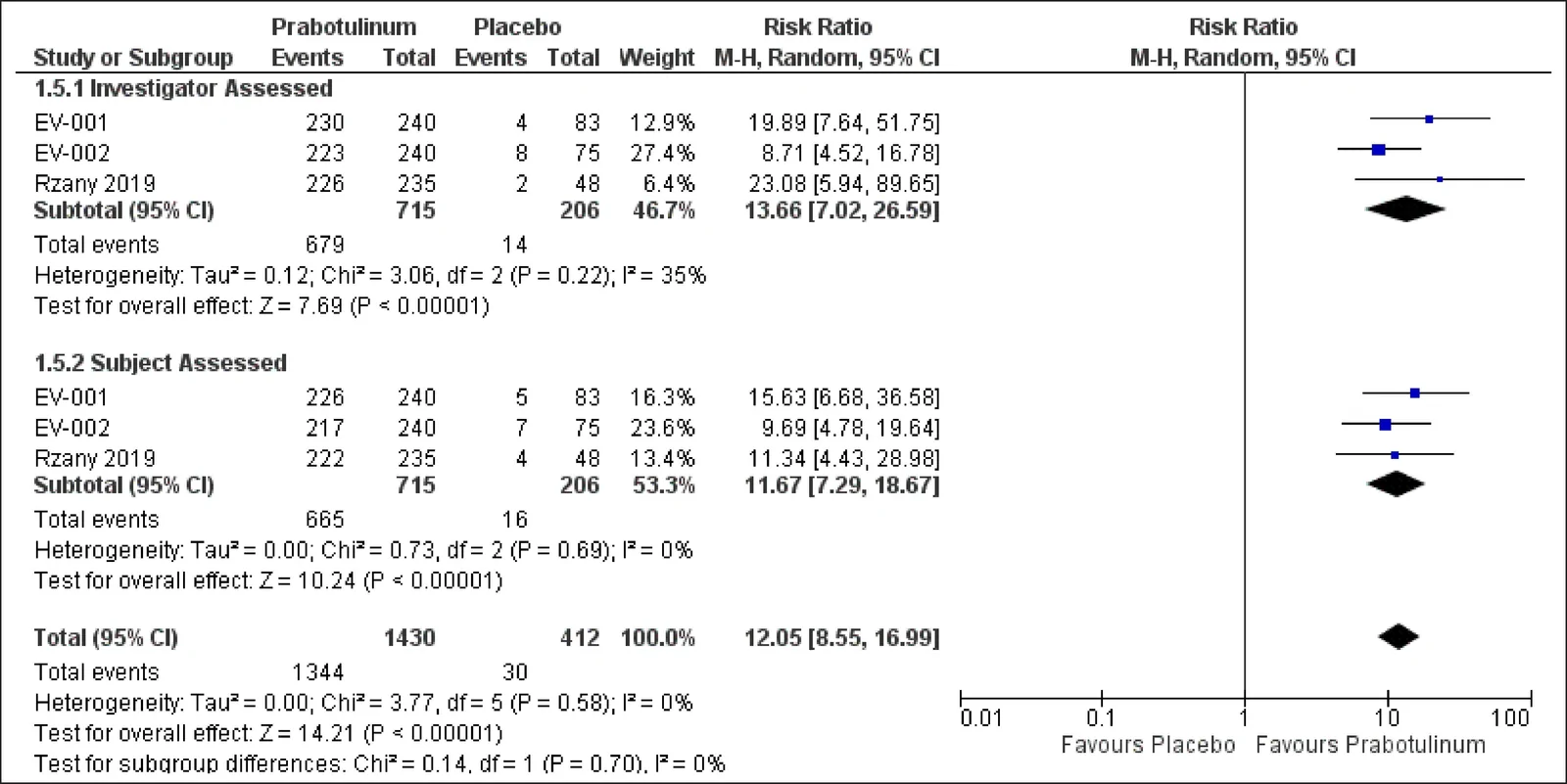
Forest plot of Global Aesthetic Improvement Scale responder rates at Day 30.

#### SSS “Satisfied” or “Very Satisfied” at Day 30

Patient-reported satisfaction, assessed through the SSS, was significantly higher in participants receiving prabotulinum (RR = 14.07, 95% CI, 8.32-23.80; *I*^2^ = 0%; *P* < .00001), with no heterogeneity across the 3 included trials (*I*^2^ = 0%), as shown in Supplementary Figure 7.

### Safety Outcomes

All 3 trials (EV-001, EV-002, and Rzany 2019) reported safety data for prabotulinum and placebo.^3,11^ No statistically significant difference was observed between groups for any prespecified safety endpoint, including overall adverse events, drug-related AESI, headache, eyelid ptosis, and TEAEs.

Overall adverse events were comparable between groups (RR = 1.14, 95% CI, 0.91-1.43; *I*^2^ = 0%; *P* = .27). Drug-related AESI were numerically higher in the prabotulinum arm but did not reach statistical significance, (RR = 2.49, 95% CI, 0.49-13.40; *I*^2^ = 0%; *P* = .29). Headache rates were similar between groups (RR = 0.92, 95% CI, 0.62-1.37; *I*^2^ = 0%; *P* = .68), and eyelid ptosis was rare and not statistically associated with treatment (RR = 1.92, 95% CI, 0.35-10.60; *I*^2^ = 0%; *P* = .45). TEAEs did not differ significantly between arms (RR = 1.43, 95% CI, 0.82-2.48; *I*^2^ = 19%; *P* = .20), as illustrated in Supplementary Figures 8-12.

Safety data from 3 additional trials not eligible for pooled quantitative analysis (EV-006, EV-004, and EV-201) are presented in Table 2.^12-14^

**Table 2:** Summary of Safety Outcomes Across PrabotulinumtoxinA Clinical Trials (EV-004, EV-006, and EV-201)

Safety parameters	Lorenc 2021 (EV-006)	Janette 2021 (EV-004)	Fagien 2024 (EV-201)
Study design	Open-label, multicenter, nonrandomized, repeat dose, Phase II safety study	Open-label, multicenter, nonrandomized, repeat dose, Phase II safety study	Multicenter, randomized, double-blind, active-controlled, Phase II
Doses of prabotulinum	20 U	20 U	20 U
Follow-up duration (days)	365	365	Up to 365 days or return to baseline
Sample size (*N*)	570	352	52
All AEs, *n*/*N* (%)	235/570 (41.2)	148/352 (42)	8/52 (15.3)
Any serious AE, *n*/*N* (%)	7/570 (1.2)	7/352 (2)	0/52 (0)
Any study drug-related AE, *n*/*N* (%)	61/570 (10.7)	51/352 (14.5)	3/52 (5.7)
Any AE leading to study discontinuation, *n*/*N* (%)	1/570 (0.2)	2/352 (0.6)	0/52 (0)
Any AE leading to death, *n*/*N* (%)	1/570 (0.2)	0/352 (0)	NR
Headache, *n*/*N* (%)	75/570 (13.2)	54/352 (15.3)	2/52 (3.8)
All AESIs, *n/N* (%)	16/570 (2.8)	11/352 (3.1)	4/52 (7.7)
Eye disorders, *n*/*N* (%)	10/570 (1.8)	7/352 (2)	NR
Eyebrow/eyelid ptosis, *n*/*N* (%)	9/570 (1.6)	5/352 (1.4)	0/52 (0)
Blepharospasm, *n*/*N* (%)	2/570 (0.4)	1/352 (0.3)	NR

AE, adverse event; AESI, adverse event of special interest; NR, not reported; *N*, number of participants; *n*, number of patients at each level of summarization; U, units; TEAE, treatment-emergent adverse event; SAE, serious adverse event.

### Certainty of Evidence

The certainty of evidence ranged from very low to moderate across the assessed outcomes. Moderate-certainty evidence indicated that prabotulinumtoxinA improved the likelihood of achieving a ≥1-point improvement in the Glabellar Line Scale (GLS) at Day 2, whereas the certainty of evidence for any adverse events and headache was also moderate. Certainty was downgraded primarily because of imprecision and concerns regarding potential publication bias because of all trials being funded from the same industry source. The certainty of evidence for GLS 0 or 1 at Day 30 and ≥1-point GLS improvement at day 150 was low, reflecting imprecision and/or concerns regarding publication bias and inconsistency. Evidence for the composite ≥2-point GLS improvement at Day 30 was very low certainty because of very serious imprecision and potential publication bias, whereas evidence for the corresponding outcome at Day 150 was low certainty owing to very serious imprecision. The GRADE profile is presented in Supplementary Table 2.

## DISCUSSION

Overall, this meta-analysis of 3 randomized, double-blind, placebo-controlled Phase III trials from the prabotulinumtoxinA clinical development program demonstrated consistently greater improvement in glabellar line severity compared with placebo across the evaluated efficacy outcomes. Participants receiving prabotulinumtoxinA were more likely to achieve none-or-mild glabellar line severity (GLS 0 or 1) at Day 30, demonstrate an early response as early as Day 2, and maintain treatment effects through Day 150. Consistent effects were observed across investigator and SA endpoints, composite responder analyses, and global aesthetic improvement measures. However, these findings should be interpreted in the context of the limited and highly homogeneous evidence base: all quantitatively synthesized trials originated from the same clinical development program and were conducted during a similar period, limiting the independence and generalizability of the available evidence. The pooled estimates therefore primarily provide a more precise summary of the available trial data rather than independent confirmation from diverse clinical research settings.

The clinical efficacy of prabotulinumtoxinA is mechanistically explained by its action as a botulinum neurotoxin type A, which selectively cleaves SNAP-25 within the presynaptic SNARE complex, thereby preventing vesicular fusion and blocking acetylcholine release at the neuromuscular junction.^24,25^ This results in reversible chemodenervation of the corrugator supercilia, procerus muscles, and depressor supercilli, leading to a reduction in repetitive muscular activity responsible for the formation of dynamic glabellar rhytids.^3^ The consequent decrease in muscle contraction permits relaxation of overlying cutaneous structures and attenuation of mechanically induced dermal folding, thereby underlying the observed clinical improvement in glabellar line severity.^26^ The onset and duration of effect reflect the gradual recovery of neuromuscular transmission through axonal sprouting and regeneration of synaptic function following restoration of SNAP-25-mediated activity.^27^ Although a prolonged reduction in repetitive muscular contraction may also reduce mechanical stress on the overlying skin; however, evidence for structural dermal remodeling in this indication remains limited.

The efficacy findings of the present meta-analysis are consistent with previously published evidence on BoNT-A in aesthetic indications, which has consistently demonstrated significantly higher responder rates compared with placebo, with peak efficacy typically observed at approximately Day 30 and sustained clinical benefit for several months.^28-30^ Direct comparisons between BoNT-A products remain limited by differences in product formulation, biological activity, and unit potency. Importantly, potency units assigned to different BoNT products are product-specific and are not considered interchangeable. Preclinical comparative data have demonstrated differences in unit-to-unit biological activity between prabotulinumtoxinA and onabotulinumtoxinA, highlighting the limitations of interpreting nominal dose units as equivalent across products. Consequently, comparisons based solely on numerical dose may be misleading, and direct comparative trials remain preferable for determining relative clinical efficacy and duration of effect.^31^

An additional finding was the consistent difference in effect magnitude between investigator- and SA outcomes. Across several efficacy analyses, investigator-assessed response estimates were larger than those based on subject assessment. This pattern was observed for GLS and global aesthetic improvement outcomes. Several factors may contribute to this discrepancy, including differences in assessment perspective, response thresholds, and the standardized training and assessment conditions used by investigators. Investigator assessments may be more standardized across participants, whereas subjects evaluate changes according to their own perception of facial appearance and may apply different thresholds for meaningful improvement. Importantly, both assessment approaches generally favored prabotulinumtoxinA, indicating consistency in the direction of treatment effect despite differences in effect magnitude. Nevertheless, the systematic difference in magnitude suggests that pooled estimates should be interpreted with attention to the assessor used and should not be assumed to represent interchangeable measures of treatment response.

The safety findings are likewise consistent with the established safety profile of BoNT-A, with previous systematic reviews demonstrating no clinically meaningful differences in overall adverse event rates between active treatment and placebo and a low incidence of treatment-related complications.^29^ In the present analysis, similar findings were observed, with no statistically significant differences detected between groups for overall adverse events, TEAEs, headache, or eyelid ptosis, and most events being mild and transient in nature.^28,29^ These observations are further supported by extended safety data from Phase II and long-term studies (EV-004, EV-006, and EV-201), in which overall adverse event rates ranged from 14.5% to 42.0%, serious adverse events remained low (0%-2.0%), and adverse events leading to discontinuation were rare (≤0.6%).^12-14^ Treatment-related adverse events occurred in ∼4.8% to 14.5% of participants, and ptosis rates remained low (0.9%-1.6%), consistent with known on-target effects of BoNT-A.^13^ Within the available evidence, these findings suggest a consistent safety profile across BoNT-A formulations and aesthetic facial indications.^28-30^

Across the included randomized controlled trials, methodological quality was generally favorable, with 2 studies (EV-001 and EV-002) judged to be at low risk of bias across key domains, including randomization, allocation concealment, blinding, incomplete outcome data, and selective reporting, whereas Rzany 2019 was judged to have some concerns overall.^3,11^ In addition, the pooled analyses demonstrated low statistical heterogeneity for the majority of efficacy outcomes, with *I*^2^ values generally close to zero, indicating a high degree of consistency in treatment effects across studies. Where moderate heterogeneity was observed in selected time point or subgroup analyses, this was reduced after sensitivity analyses without meaningful changes in overall effect estimates. The consistency of effect estimates across investigator- and SA outcomes further supports the reliability of the observed treatment benefit. Although the included trials were generally well conducted and demonstrated consistent treatment effects, the interpretation of the pooled estimates should remain cautious given the small number of trials, their substantial methodological similarity, and their origin from the same clinical development program. Low statistical heterogeneity therefore indicates consistency among the available trials but should not be interpreted as evidence of broad reproducibility across independent study populations or research settings.

### Limitations

This meta-analysis has several limitations that should be acknowledged. First, the evidence base comprises a limited number of randomized controlled trials, which may reduce the precision of pooled effect estimates and limit the scope of subgroup analyses. Second, although the included studies were generally at low risk of bias, variability in study design, dosing protocols, and outcome definitions may contribute to clinical heterogeneity not fully captured by statistical measures. Third, the duration of follow-up for primary efficacy outcomes was relatively short, limiting assessment of long-term durability beyond the study observation periods. Fourth, safety evidence was supplemented by Phase II and open-label studies, which are subject to inherent methodological limitations, including potential selection and reporting bias. Finally, the absence of direct head-to-head comparisons with other BoNT-A formulations limits definitive comparative conclusions regarding relative efficacy and safety. An important limitation is that all 3 trials contributing to the quantitative analysis originated from the same prabotulinumtoxinA clinical development program and were conducted within a relatively narrow period, with similar study designs and populations. Consequently, the apparent consistency across trials should not be interpreted as equivalent to replication across independent research groups or sponsors. Moreover, the industry-sponsored nature of the evidence base introduces the possibility of sponsorship-related bias and limits the extent to which the findings can be generalized beyond the populations and conditions represented in the pivotal development program. Although pooling these trials improves statistical precision, it does not overcome the limited independence or breadth of the underlying evidence base.

## CONCLUSIONS

The findings of this meta-analysis suggest that prabotulinumtoxinA is well-tolerated, rapid-acting, and effective across multiple clinician-reported and patient-reported outcomes for the treatment of moderate-to-severe glabellar lines. No statistically significant increase in adverse events was observed compared with placebo. However, further large-scale studies with longer follow-up durations and direct comparative trials with other BoNT formulations are required to better define the long-term clinical role and comparative effectiveness of prabotulinumtoxinA in aesthetic dermatology.

## Supplementary Material

ojag202_Supplementary_Data

## References

[1] Klassen AF, Cano SJ, Schwitzer JA, Scott AM, Pusic AL. Development and psychometric validation of the FACE-Q skin, lips, and facial rhytids appearance scales and adverse effects checklists for cosmetic procedures. JAMA Dermatol. 2016;152:443–451. doi: 10.1001/jamadermatol.2016.0018 PMC4833666 26934294

[2] Cox SE, Kaufman-Janette J, Cohen JL, et al Letibotulinumtoxina attenuates the psychological burden of glabellar lines and is associated with high subject satisfaction in phase 3 clinical trials. Dermatol Surg. 2024;50:535–541. doi: 10.1097/DSS.0000000000004152 38470985

[3] Beer KR, Shamban AT, Avelar RL, Gross JE, Jonker A. Efficacy and safety of prabotulinumtoxinA for the treatment of glabellar lines in adult subjects: results from 2 identical phase III studies. Dermatol Surg. 2019;45:1381–1393. doi: 10.1097/DSS.0000000000001903 PMC6819028 30893162

[4] Li X, Sui C, Xia X, Chen X. Efficacy and safety of botulinum toxin type A for treatment of glabellar lines: a network meta-analysis of randomized controlled trials. Aesthetic Plast Surg. 2023;47:365–377. doi: 10.1007/s00266-022-03018-y 36097079

[5] Lemdani MS, Honig SE, Habarth-Morales TE, et al Comparison of botulinum toxin A formulations for glabellar strain treatment in women: a double-blind randomized clinical trial. JAMA Dermatol. 2025;161:723–730. doi: 10.1001/jamadermatol.2025.1335. Erratum in: JAMA Dermatol. 2025 Aug 20. doi: 10.1001/jamadermatol.2025.3297 PMC12120680 40434770

[6] Matak I, Bölcskei K, Bach-Rojecky L, Helyes Z. Mechanisms of botulinum toxin type A action on pain. Toxins (Basel). 2019;11:459. doi: 10.3390/toxins11080459 PMC6723487 31387301

[7] Carruthers J, Carruthers A, Blitzer A, Eadie N, Brin MF. Treatment of glabellar lines with Botox (onabotulinumtoxinA): development, insights, and impact. Medicine (Baltimore). 2023;102:e32375. doi: 10.1097/MD.0000000000032375 PMC10374180 37499082

[8] Nestor MS, Ablon GR. Comparing the clinical attributes of abobotulinumtoxinA and onabotulinumtoxinA utilizing a novel contralateral Frontalis model and the Frontalis Activity Measurement Standard. J Drugs Dermatol. 2011;10:1148–1157. 21968665

[9] Guo Y, Lu Y, Liu T, et al Efficacy and safety of botulinum toxin type A in the treatment of glabellar lines: a meta-analysis of randomized, placebo-controlled, double-blind trials. Plast Reconstr Surg. 2015;136:310e–318e. doi: 10.1097/PRS.0000000000001544 26313835

[10] Evolus, Inc. JEUVEAU® (prabotulinumtoxinA-xvfs) for injection, for intramuscular use: prescribing information. Newport Beach, CA: Evolus, Inc.; revised April 2023. Available from: https://image.newsletter.evolus.com/lib/fe3511737164047c751277/m/1/7a8b57cf-e5b3-4e8f-99ab-6f9ab170cbe5.pdf

[11] Rzany B, Ascher B, Fratila A, et al A multicenter, randomized, double-blind, placebo-controlled, single-dose, phase III, non-inferiority study comparing prabotulinumtoxinA and onabotulinumtoxinA for the treatment of moderate to severe glabellar lines in adult patients. Aesthet Surg J. 2020;40:413–429. doi: 10.1093/asj/sjz110 30951166

[12] Fagien S, Avelar RL, Cox SE, Joseph JH, Kaufman-Janette J, Marcus KA. Safety and duration of effect of 40-unit prabotulinumtoxinA-xvfs for the treatment of moderate to severe glabellar lines in adult patients: a phase II, multicenter, randomized, double-blind, active-controlled trial. Aesthet Surg J. 2024;44:987–1000. doi: 10.1093/asj/sjae051 PMC11334208 38506148

[13] Kaufman-Janette J, Fagien S, Biesman BS, et al The first of two one-year, multicenter, open-label, repeat-dose, phase II safety studies of prabotulinumtoxinA for the treatment of moderate to severe glabellar lines in adult patients. Aesthet Surg J. 2021;41:1409–1422. doi: 10.1093/asj/sjaa383 PMC8598194 33944905

[14] Lorenc ZP, Kaufman-Janette J, Kenkel JM, et al The second of two one-year, multicenter, open-label, repeat-dose, phase II safety studies of prabotulinumtoxinA for the treatment of moderate to severe glabellar lines in adult patients. Aesthet Surg J. 2021;41:1423–1438. doi: 10.1093/asj/sjaa382 PMC8598184 33944913

[15] Gadarowski MB, Ghamrawi RI, Taylor SL, Feldman SR. PrabotulinumtoxinA-xvfs for the treatment of moderate-to-severe glabellar lines. Ann Pharmacother. 2021;55:354–361. doi: 10.1177/1060028020943527 32698599

[16] Page MJ, McKenzie JE, Bossuyt PM, et al The PRISMA 2020 statement: an updated guideline for reporting systematic reviews. BMJ. 2021;372:n71. doi: 10.1136/bmj.n71 PMC8005924 33782057

[17] Shahid Zain Ul Abideen, Fatima Syeda Huda, Faisal Mehak, et al Efficacy and safety of prabotulinumtoxinA for the treatment of moderate to severe glabellar lines: a GRADE assessed systematic review and meta-analysis. PROSPERO 2026 CRD420261394860 [Internet]. University of York; 2026. Cited June 16, 2026. https://www.crd.york.ac.uk/PROSPERO/view/CRD420261394860.

[18] Ouzzani M, Hammady H, Fedorowicz Z, Elmagarmid A. Rayyan—a web and mobile app for systematic reviews. Syst Rev. 2016;5:210. doi: 10.1186/s13643-016-0384-4 PMC5139140 27919275

[19] Amir-Behghadami M, Janati A. Population, Intervention, Comparison, Outcomes and Study (PICOS) design as a framework to formulate eligibility criteria in systematic reviews. Emerg Med J. 2020;37:387. doi: 10.1136/emermed-2020-209567 32253195

[20] Sterne JAC, Savović J, Page MJ, et al Rob 2: a revised tool for assessing risk of bias in randomised trials. BMJ. 2019;366:l4898. doi: 10.1136/bmj.l4898 31462531

[21] Schmidt L, Shokraneh F, Steinhausen K, Adams CE. Introducing RAPTOR: RevMan Parsing Tool for reviewers. Syst Rev. 2019;8:151. doi: 10.1186/s13643-019-1070-0 PMC6595567 31242929

[22] DerSimonian R, Laird N. Meta-analysis in clinical trials. Control Clin Trials. 1986;7:177–188. doi: 10.1016/0197-2456(86)90046-2 3802833

[23] Brozek JL, Canelo-Aybar C, Akl EA, et al GRADE guidelines 30: the GRADE approach to assessing the certainty of modeled evidence—an overview in the context of health decision-making. J Clin Epidemiol. 2021;129:138–150. doi: 10.1016/j.jclinepi.2020.09.018 PMC8514123 32980429

[24] Pirazzini M, Rossetto O, Eleopra R, Montecucco C. Botulinum neurotoxins: biology, pharmacology, and toxicology. Pharmacol Rev. 2017;69:200–235. doi: 10.1124/pr.116.012658 PMC5394922 28356439

[25] Rossetto O, Pirazzini M, Montecucco C. Botulinum neurotoxins: genetic, structural and mechanistic insights. Nat Rev Microbiol. 2014;12:535–549. doi: 10.1038/nrmicro3295 24975322

[26] Yi KH, Lee JH, Hu HW, Kim HJ. Anatomical proposal for botulinum neurotoxin injection for glabellar frown lines. Toxins (Basel). 2022;14:268. doi: 10.3390/toxins14040268 PMC9032255 35448877

[27] Meunier FA, Schiavo G, Molgó J. Botulinum neurotoxins: from paralysis to recovery of functional neuromuscular transmission. J Physiol Paris. 2002;96:105–113. doi: 10.1016/S0928-4257(01)00086-9 11755789

[28] Naumann M, Jankovic J. Safety of botulinum toxin type A: a systematic review and meta-analysis. Curr Med Res Opin. 2004;20:981–990. doi: 10.1185/030079904125003962 15265242

[29] Camargo CP, Xia J, Costa CS, et al Botulinum toxin type A for facial wrinkles. Cochrane Database Syst Rev. 2021;7:CD011301. doi: 10.1002/14651858.CD011301.pub2 PMC8407355 34224576

[30] Cavallini M, Cirillo P, Fundarò SP, et al Safety of botulinum toxin A in aesthetic treatments: a systematic review of clinical studies. Dermatol Surg. 2014;40:525–536. doi: 10.1111/dsu.12463 24575858

[31] Rupp DC, Canty D, Rheaume C, et al A preclinical study comparing the activity and potency of onabotulinumtoxinA and prabotulinumtoxinA. Clin Cosmet Investig Dermatol. 2023;16:581–591. doi: 10.2147/CCID.S397999 PMC10008670 36923693

