# Supplementary material for: Efficacy and Safety of PrabotulinumtoxinA for the Treatment of Moderate-to-Severe Glabellar Lines: A GRADE-Assessed Systematic Review and Meta-Analysis
Source: Aesthet Surg J Open Forum. 2026 Oct 14;8:ojag202. doi: 10.1093/asjof/ojag202 (PMC13760622; doi:10.1093/asjof/ojag202)
Supplement: Supplementary file 1 [file ojag202_supplementary_data.docx]

**Supplementary Table 1:** Detailed search strategies used for each database

| **DATABASE** | **SEARCH STRATEGY** | **SEARCH RESULTS** |
| --- | --- | --- |
| **Pubmed** | ("Botulinum Toxins, Type A"[Mesh] OR "Botulinum Toxins"[Mesh] OR prabotulinumtoxinA[tiab] OR "prabotulinumtoxinA-xvfs"[tiab] OR prabotulinum[tiab] OR Jeuveau[tiab] OR "botulinum toxin type A"[tiab] OR "botulinum toxin A"[tiab])   AND   ("Glabellar Lines"[tiab] OR "glabellar line*"[tiab] OR "glabellar wrinkle*"[tiab] OR "facial wrinkle*"[tiab] OR "frown line*"[tiab] OR "glabellar rhytid*"[tiab] OR "moderate to severe glabellar lines"[tiab])   AND   ("Placebos"[Mesh] OR placebo[tiab] OR comparator[tiab] OR control*[tiab]) | 162 |
| **Clinical trial gov** | **Condition/disease:** "Glabellar Lines" OR "Glabellar Wrinkles" OR "Facial Wrinkles" OR "Frown Lines"  **Intervention/treatment:** prabotulinumtoxinA OR "prabotulinumtoxinA-xvfs" OR Jeuveau OR "botulinum toxin type A" | 136 |
| **Cochrane** | #1 "glabellar line*" OR "glabellar wrinkle*" OR "facial wrinkle*" OR "frown line*" OR "glabellar rhytid*" OR "moderate to severe glabellar lines"   #2 prabotulinumtoxinA OR "prabotulinumtoxinA-xvfs" OR prabotulinum OR Jeuveau OR "botulinum toxin type A"   #3 placebo OR comparator OR control*  #4 #1 AND #2 AND #3 | 320 |
| **Scopus** | TITLE-ABS-KEY("glabellar line*" OR "glabellar wrinkle*" OR "facial wrinkle*" OR "frown line*" OR "glabellar rhytid*" OR "moderate to severe glabellar lines")   AND   TITLE-ABS-KEY(prabotulinumtoxinA OR "prabotulinumtoxinA-xvfs" OR prabotulinum OR Jeuveau OR "botulinum toxin type A")   AND   TITLE-ABS-KEY(placebo OR comparator OR control* OR "onabotulinumtoxinA" OR Botox) | 363 |
| **Embase** | #1 'botulinum toxin type a'/exp OR 'botulinum toxin'/exp OR prabotulinumtoxina:ti,ab OR 'prabotulinumtoxina-xvfs':ti,ab OR prabotulinum:ti,ab OR jeuveau:ti,ab OR 'botulinum toxin type a':ti,ab   #2 'glabellar line':ti,ab OR 'glabellar wrinkle':ti,ab OR 'facial wrinkle':ti,ab OR 'frown line':ti,ab OR 'glabellar rhytid':ti,ab OR 'moderate to severe glabellar lines':ti,ab   #3 'placebo'/exp OR placebo:ti,ab OR comparator:ti,ab OR control*:ti,ab OR 'onabotulinumtoxina':ti,ab OR botox:ti,ab   #4 #1 AND #2 AND #3 | 224 |

**Supplementary Table 2.** GRADE Assessment

| **Certainty assessment** | | | | | | | **Summary of findings** | | | | |
| --- | --- | --- | --- | --- | --- | --- | --- | --- | --- | --- | --- |
| **Participants  (studies)  Follow-up** | **Risk of bias** | **Inconsistency** | **Indirectness** | **Imprecision** | **Publication bias** | **Overall certainty of evidence** | **Study event rates (%)** | | **Relative effect  (95% CI)** | **Anticipated absolute effects** | |
|  |  |  |  |  |  |  | **With placebo** | **With Efficacy Outcomes** |  | **Risk with placebo** | **Risk difference with Efficacy Outcomes** |
| **GLS 0 or 1 at day 30** | | | | | | | | | | | |
| 1886  (3 RCTs) | not serious | not serious | not serious | serious^a^ | publication bias suspected^b^ | ⨁⨁◯◯  Low^a,b^ | 17/420 (4.0%) | 1227/1466 (83.7%) | RR 19.24  (12.08 to 30.64) | 17/420 (4.0%) | 738 more per 1,000  (from 448 more to 1,000 more) |
| **≥1-point GLS improvement at day 2** | | | | | | | | | | | |
| 1565  (3 RCTs) | not serious | not serious | not serious | not serious | publication bias suspected^c^ | ⨁⨁⨁◯  Moderate^c^ | 49/365 (13.4%) | 605/1200 (50.4%) | RR 3.61  (2.52 to 5.19) | 49/365 (13.4%) | 350 more per 1,000  (from 204 more to 562 more) |
| **≥1-point GLS improvement at day 150** | | | | | | | | | | | |
| 1551  (3 RCTs) | not serious | serious^d^ | not serious | not serious | publication bias suspected^e^ | ⨁⨁◯◯  Low^d,e^ | 43/364 (11.8%) | 466/1187 (39.3%) | RR 3.47  (2.13 to 5.65) | 43/364 (11.8%) | 292 more per 1,000  (from 133 more to 549 more) |
| **Composite ≥2-point GLS improvement at day 30 - Investigator + Subject Assessed** | | | | | | | | | | | |
| 638  (2 RCTs) | not serious | not serious | not serious | very serious^f^ | publication bias suspected^g^ | ⨁◯◯◯  Very low^f,g^ | 2/158 (1.3%) | 331/480 (69.0%) | RR 54.39  (13.71 to 215.86) | 2/158 (1.3%) | 676 more per 1,000  (from 161 more to 1,000 more) |
| **Composite ≥2-point GLS improvement (IA+SA) - At Day 150** | | | | | | | | | | | |
| 632  (2 RCTs) | not serious | not serious | not serious | very serious^h^ | none | ⨁⨁◯◯  Low^h^ | 0/158 (0.0%) | 22/474 (4.6%) | RR 7.73  (1.05 to 56.77) | 0/158 (0.0%) | 0 fewer per 1,000  (from 0 fewer to 0 fewer) |
| **Any Adverse Events** | | | | | | | | | | | |
| 948  (3 RCTs) | not serious | not serious | not serious | serious^i^ | none | ⨁⨁⨁◯  Moderate^i^ | 64/211 (30.3%) | 256/737 (34.7%) | RR 1.14  (0.91 to 1.43) | 64/211 (30.3%) | 42 more per 1,000  (from 27 fewer to 130 more) |
| **Headache** | | | | | | | | | | | |
| 948  (3 RCTs) | not serious | not serious | not serious | serious^j^ | none | ⨁⨁⨁◯  Moderate^j^ | 28/211 (13.3%) | 91/737 (12.3%) | RR 0.92  (0.62 to 1.37) | 28/211 (13.3%) | 11 fewer per 1,000  (from 50 fewer to 49 more) |

**CI: confidence interval; RR: risk ratio**

Explanations

a. Downgraded one level for serious imprecision: The relatively small number of placebo events resulted in uncertainty around the precise magnitude of the treatment effect, despite the large overall effect favoring prabotulinumtoxinA.

b. Downgraded one level due to potential publication bias: only three trials were available, all from the same industry-sponsored clinical development program, and selective dissemination could not be confidently excluded.

c. Downgraded one level due to potential publication bias: only three trials were available, all from the same industry-sponsored clinical development program, and selective dissemination could not be confidently excluded.

d. Downgraded one level for serious inconsistency: Although the direction of effect was consistent, there was variability in the magnitude and reporting of effects across studies, with this outcome reported as a secondary endpoint in Rzany et al. but as an exploratory endpoint in EV-001 and EV-002.

e. Downgraded one level due to potential publication bias: only three trials were available, all from the same industry-sponsored clinical development program, and selective dissemination could not be confidently excluded.

f. Very serious imprecision: Downgraded two levels because the confidence interval around the pooled effect is very wide (RR 54.39, 95% CI 13.71–215.86), indicating substantial uncertainty in the magnitude of the treatment effect.

g. Downgraded one level due to potential publication bias: only three trials were available, all from the same industry-sponsored clinical development program, and selective dissemination could not be confidently excluded.

h. Very serious imprecision: Downgraded two levels because there were very few events and the confidence interval was very wide (RR 7.73, 95% CI 1.05–56.77), resulting in substantial uncertainty in the magnitude of the treatment effect.

i. Pooled RR= 1.14 (95% CI : 0.91to 1.43) crosses the line of no effect, is statistically non significant, and the CI includes both possible benefits and no benefits/ harm.

**j. Pooled RR= 0.92 (95% CI : 0.62 to 1.37) crosses the line of no effect, is statistically non significant, and the CI includes both possible benefits and no benefits/ harm.**

**Supplementary Figure 1.** Risk of bias summary of included studies.

**Supplementary Figure 2.** Risk of bias graph of included studies.

**Supplementary Figure 3.** Sensitivity analysis of ≥1-point GLS improvement at Day 2 after exclusion of EV-001.

**Supplementary Figure 4.** Sensitivity analysis of ≥1-point GLS improvement at Day 150 after exclusion of EV-002.

**Supplementary Figure 5.** Forest plot of ≥2-point GLS improvement at Day 30.

**Supplementary Figure 6.** Forest plots of composite investigator- and subject-assessed ≥2-point GLS improvement at Days 90, 120, and 150.

**Supplementary Figure 7.** Forest plot of Subject Satisfaction Scale (SSS) responder rates at Day 30.

**Supplementary Figure 8.** Forest plot of overall adverse events.

**Supplementary Figure 9.** Forest plot of drug-related adverse events of special interest.

**Supplementary Figure 10.** Forest plot of headache incidence.

**Supplementary Figure 11.** Forest plot of eyelid ptosis.

**Supplementary Figure 12.** Forest plot of treatment-emergent adverse events.
